# Mercury and Depletion in the Fevers of India

**Published:** 1831

**Authors:** 


### Mercury and Depletion in the Fevers of India.

In a very interesting and highly practical work on the climate, diseases, and topography of Pulo Penang, by Dr. Ward and Mr. Grant, we find the following statement, which shews that so late as the year 1830, the efficacy of the mercurial and depletive practice in Indian diseases, has lost nothing of its influence, notwithstanding the lachrymose ululations of certain old women in this country.

" In the European Patient depletion to a large extent is positively called for to save life: it therefore is a necessary evil. But in the native from his habits of body assimilating him more to these climes, the blood, which in him is always thinner than it is in the European, and which appears to be not so easily disorganized by noxious inhalations, can flow through all the extreme vessels without producing dangerous congestion, and that to such an extent only as can be removed without depletion, by means of antimonials, mercurials, and counter-irritants.

*Mercury.* In a brief way, touching upon the wonderful influence that Mercury (in whatever form administered so as to affect the system) exerts in putting an immediate check to this, as well as to all other acute intertropical maladies, I was induced, by perusing the works of that enlightened and elegant writer, Dr. James Johnson, who, it must be allowed, is the progenitor of the present active and extended system pursued in the treatment of tropical diseases, to be one of the happy followers of his doctrine. And it is to be hoped that all who follow his precepts will have equal gratification and success therefrom. In the first place, then, this medicine, whenever it produces a healthy action in the glands, more particularly in the Liver, subdues this fever; but extended practice must point out, that, in acute diseases, this rarely happens *till ptyalism is established,*—when the petroleum or tarlike dejections indicate that the gall-bladder has gorged forth its long inspissated and diseased tenant. There is little further use for its influence excepting to keep up a gentle action in the glands. The case of Lieut. C. is the only one in which I have seen life saved, in this fever, without its being through the medium of mercury carried to the length of ptyalism; and I look upon it to be a matter of the most essential importance to the welfare of our fellow sojourners in India, that this fact should be particularly enquired into. Facts are stubborn things; and I uphold my opinions on them alone. Some medical men suppose its influence is not required. I will conclude my remarks upon it with the words of that celebrated practitioner Dr. Chisholm on the administration of it in the malignant pestilential fever, p. 221. 'Are we then from vain and unfounded apprehensions of this kind, from reasoning drawn from false premises, or from the suggestions of uninformed or prejudiced minds, to yield up the result of our own frequently reiterated experience, to relinquish the best aid we can bring to the relief and support of our fellow creatures suffering under so direful a malady? forbid it humanity! forbid it truth! forbid it heaven!'"

From the foregoing extract it will be seen that certain anathemas thundered forth against mercury in the dangerous diseases of India, are but little regarded by those practitioners who can see with their own eyes, and are capable of drawing useful deductions from solid facts.

---

